# Redefining MRI-Based Skull Segmentation Through AI-Driven Multimodal Integration

**DOI:** 10.3390/jimaging11110372

**Published:** 2025-10-22

**Authors:** Michel Beyer, Alexander Aigner, Alexandru Burde, Alexander Brasse, Sead Abazi, Lukas B. Seifert, Jakob Wasserthal, Martin Segeroth, Mohamed Omar, Florian M. Thieringer

**Affiliations:** 1 Department of Oral and Cranio-Maxillofacial Surgery and 3D Print Lab, University Hospital Basel, 4031 Basel, Switzerland; 2 Medical Additive Manufacturing Research Group (Swiss MAM), Department of Biomedical Engineering, University of Basel, 4123 Allschwil, Switzerland; 3 Sarcoma Centre, Hannover Medical School, 30625 Hannover, Germany; 4 Department of Dental Technology, Faculty of Nursing and Life Sciences, Iuliu Hatieganu University of Medicine and Pharmacy, 400012 Cluj-Napoca, Romania; 5 Clinic of Radiology and Nuclear Medicine, University Hospital Basel, 4031 Basel, Switzerland

**Keywords:** artificial intelligence, segmentation, magnetic resonance imaging, computed tomography, personalized medicine

## Abstract

Skull segmentation in magnetic resonance imaging (MRI) is essential for cranio-maxillofacial (CMF) surgery planning, yet manual approaches are time-consuming and error-prone. Computed tomography (CT) provides superior bone contrast but exposes patients to ionizing radiation, which is particularly concerning in pediatric care. This study presents an AI-based workflow that enables skull segmentation directly from routine MRI. Using 186 paired CT–MRI datasets, CT-based segmentations were transferred to MRI via multimodal registration to train dedicated deep learning models. Performance was evaluated against manually segmented CT ground truth using Dice Similarity Coefficient (DSC), Mean Surface Distance (MSD), and Hausdorff Distance (HD). AI achieved higher performance on CT (DSC 0.981) than MRI (DSC 0.864), with MSD and HD also favoring CT. Despite lower absolute accuracy on MRI, the approach substantially improved segmentation quality compared with manual MRI methods, particularly in clinically relevant regions. This automated method enables accurate skull modeling from standard MRI without radiation exposure or specialized sequences. While CT remains more precise, the presented framework enhances MRI utility in surgical planning, reduces manual workload, and supports safer, patient-specific treatment, especially for pediatric and trauma cases.

## 1. Introduction

Reconstructive procedures in cranio-maxillofacial (CMF) surgery pose significant challenges due to the intricate three-dimensional spatial relationships among vital anatomical structures. The primary objective is to achieve both functionally satisfactory outcomes, in terms of speech, mastication, swallowing, breathing, and facial animation, as well as aesthetically pleasing results [1,2].

Virtual Surgical Planning (VSP) and three-dimensional (3D)-printed surgical models have emerged as powerful tools to aid surgeons in overcoming these challenges. By providing quantitative data on parameters such as bone thickness, anatomical distances, and volume, these technologies can guide osteotomies, improve the precision of hardware positioning, and enhance fixation techniques [1]. Additionally, virtual surgical planning (VSP) provides a more comprehensive understanding of the surgical field, allows surgeons to simulate procedural steps in advance, and streamlines intraoperative workflows. In this context, 3D-printed anatomical models play a pivotal role by supporting detailed preoperative planning. Although direct pre-bending of metal plates prior to surgery is restricted under current medical device regulations, these models facilitate faster and more accurate intraoperative adaptation, thereby reducing operative time and improving surgical outcomes [1,2,3].

High-resolution computed tomography (CT) imaging is considered the gold standard for generating data for VSP and 3D model creation [1,2,3]. Once acquired, the imaging data can be processed and segmented either by in-house teams or by external service providers, resulting in a virtual three-dimensional reconstruction of the skeletal structures [1]. The simplest segmentation approach, thresholding, relies on intensity differences, typically using predefined Hounsfield Unit (HU) values. For example, bone is usually identified between 250 and 3000 HU, while air is around −1000 HU [2]. The widespread availability of CT scans supports the increasing adoption of these techniques, with global CT scan volume growing approximately 4% annually, reaching about 300 million scans worldwide in 2020 [4].

However, CT imaging is not without drawbacks—chief among them is radiation exposure [1,2,5]. This issue is particularly pressing in pediatric patients, where radiation can increase the risk of brain tumors, leukemia, and other malignancies over a lifetime [1,2,5]. Studies indicate that exposure to 50–60 mGy, equivalent to two or three pediatric cranial CT scans, can triple the risk of brain tumors [6].

As a radiation-free alternative, magnetic resonance imaging (MRI) holds promise, especially for young patients. MRI offers superior soft tissue contrast without ionizing radiation, enabling the depiction of critical neurovascular structures such as the inferior alveolar nerve and inferior alveolar artery, as well as detection of periapical lesions or inflammation of the dental pulp [7,8,9,10,11,12,13,14]. These benefits have led to MRI’s broader adoption in dental, orthodontic, and maxillofacial diagnostics.

Nonetheless, MRI presents its own challenges: distinguishing bone from soft tissue is technically more complex because MRI lacks predefined pixel values for tissue contrast, leading to frequent tissue overlap that complicates segmentation [15]. While newer MRI sequences such as Ultrashort Echo Time (UTE), Zero Echo Time (ZTE), or Black Bone enhance bone-to-soft-tissue contrast [16,17,18,19], they are not yet standardized in routine clinical workflows. Moreover, the presence of metal artifacts from dental restorations can severely impact image quality, complicating segmentation accuracy and reproducibility [20].

Manual segmentation remains a time-consuming task, averaging over an hour per case for expert operators [5]. This challenge is further exacerbated when using MRI data, where slice thicknesses of up to 3 mm require interpolation between slices, thereby reducing spatial accuracy and increasing the segmentation effort.

To address these limitations, researchers are actively exploring AI-based segmentation algorithms. Such algorithms could streamline the time-consuming process of manual MRI segmentation while improving consistency and accuracy [3,5,21,22,23].

The current study aims to develop an AI-based algorithm for automatic skull segmentation across standard MRI sequences. This new approach will be evaluated by comparing it to manually segmented CT data as well as AI-assisted CT segmentation results. By examining the performance, accuracy, and efficiency of AI-driven segmentation techniques, this study seeks to advance skull imaging and improve outcomes in CMF surgery.

## 2. Materials and Methods

**Figure 1:**
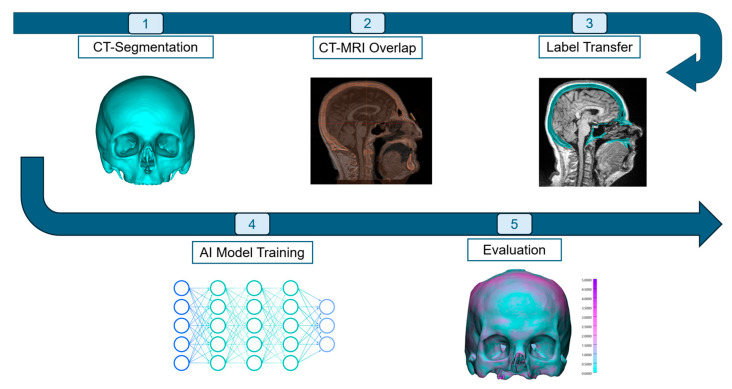
Overview of the proposed AI-based workflow for skull segmentation from magnetic resonance imaging (MRI). High-resolution skull segmentations were first obtained from computed tomography (CT) data and registered onto corresponding MRI scans through multimodal alignment. The resulting paired CT–MRI datasets were used to train a three-dimensional nnU-Net model for automatic skull segmentation directly from standard MRI sequences. The workflow includes data acquisition, CT segmentation, CT–MRI registration, label transfer, AI model training, and evaluation.

This section outlines the approach used to develop and train the artificial intelligence models for segmenting the cranium from CT and MRI scans. It includes details on the collection and preprocessing of data, the segmentation process, the training of the AI model, subsequent refinement through postprocessing, and the evaluation of model performance using validation metrics and statistical methods. The workflow of the study is displayed in Figure 1.

### 2.1. Data Acquisition

A total of 186 head and neck CT scans and their corresponding MRI images were obtained from the University Hospital Basel to support the development of the system. To ensure a representative and heterogeneous dataset, a broad spectrum of imaging modalities and acquisition protocols was included. This encompassed, among others, low-resolution PET-CT scans with slice thicknesses of up to 3.0 mm, allowing for a diverse anatomical and radiological presentation. For the CT scans, the mean number of slices was 94.2 ± 55.0, with a mean slice thickness of 1.13 ± 0.96 mm. Regarding the MRI data, the dataset comprised 78 T1-weighted, 86 T2-weighted, and 23 FLAIR sequences. On average, MRI images consisted of 107.1 ± 79.8 slices, with a mean slice thickness of 2.23 ± 1.73 mm. The magnetic field strength was 1.5 Tesla for 86 images and 3.0 Tesla for 101 images, reflecting the inclusion of scans from different clinical systems and acquisition settings. This variability in the imaging data was intentionally incorporated to develop a robust and adaptable system capable of maintaining reliable performance across a wide range of clinical imaging conditions, including scenarios involving lower image quality or non-standard acquisition protocols. To standardize the voxel size and improve the consistency of the images, all scans were resampled using the B-spline algorithm, resulting in isotropic voxels of 0.5 mm. The processed images were subsequently stored in the Digital Imaging and Communications in Medicine (DICOM) format.

### 2.2. Image Segmentation and Registration

**Figure 2:**
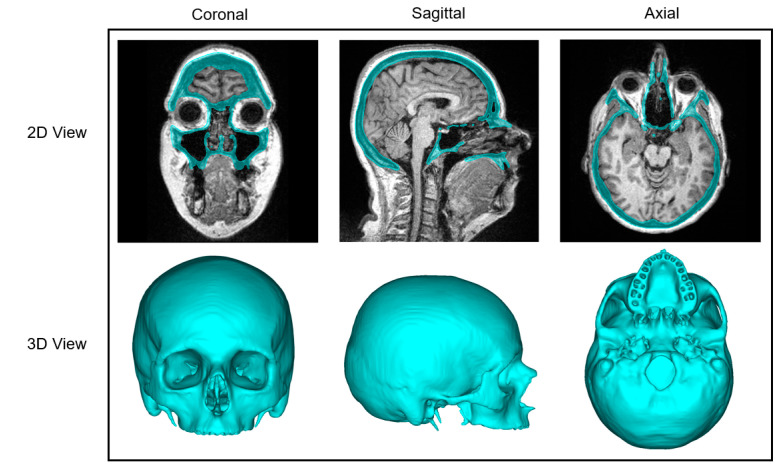
Multiplanar 2D views (coronal, sagittal, axial) and 3D reconstructions illustrating computed tomography (CT)-based skull segmentations registered onto corresponding magnetic resonance imaging (MRI) data.

The CT images were imported into Mimics Innovation Suite (Version 25.0, Materialise NV, Leuven, Belgium), where semi-automatic segmentation of the skull was performed. Following this, the corresponding MRI images were imported, and the CT images were registered and aligned with the MRI data. The transformation matrix between the two imaging modalities was saved. The segmented skull from the CT data was then transferred onto the MRI images, as shown in Figure 2. Both the CT-based skull segmentation overlaid on the CT images and the CT-based skull segmentation registered to the MRI images were exported as Standard Tessellation Language (STL) files. Finally, the STL files of the skull and the corresponding image modality were converted into two separate Neuroimaging Informatics Technology Initiative (NIfTI) files of isotropic 0.5 mm voxel size. This approach made it possible to leverage the superior quality of skull segmentations obtained from CT images, benefiting from their higher bone-to-soft-tissue contrast and enhanced image clarity. By mapping the high-precision segmentations derived from CT scans onto the corresponding MRI images, it was possible to attain a greater segmentation accuracy than would be achievable through manual segmentation of MRI data alone.

### 2.3. Dataset Generation

Two distinct datasets were created for analysis. The first dataset includes computed tomography (CT) images and their corresponding segmented skull masks overlaid directly onto the CT scans. The second dataset consists of magnetic resonance imaging (MRI) scans along with the corresponding skull segmentations originally derived from CT, also overlaid onto their respective MRI images. Both datasets were identically divided into training and test subsets, with the test subset containing approximately 15% of all images. The allocation of images into training and test sets was performed through random selection to ensure unbiased distribution. Consequently, each dataset comprised a total of 157 training images and 29 test images, together with their associated labels.

### 2.4. Assessments

Two separate artificial intelligence models were developed and trained independently: the first model utilized exclusively the CT dataset, while the second model was trained solely on the MRI dataset. The segmentation approach employed the nnU-Net framework, which automatically customizes both the training procedure and neural network architecture according to the input data characteristics [24]. Specifically, the 3d_fullres configuration was chosen, leveraging a 3D U-Net model architecture capable of processing extensive volumetric patches derived from the imaging data. In typical 3d_fullres settings, nnU-Net selects a patch size of approximately 128 × 128 × 128 voxels, paired with a batch size of 2, optimized for GPUs with around 10 GB of memory. The network topology is adapted based on the chosen patch size, with the number of pooling and upsampling stages, network depth, and feature map widths determined automatically through planning heuristics.

Training followed the default schedule of 1000 epochs. The loss function combined Dice loss with cross-entropy, and optimization was performed using stochastic gradient descent with Nesterov momentum. The models were trained on a workstation equipped with an NVIDIA RTX 4070 Ti GPU (12 GB VRAM), which closely matches the memory profile for which the default configuration is optimized.

### 2.5. Intersection Calculation

Due to differences in volume dimensions between corresponding MRI and CT images, an intersection volume was calculated for each CT-MRI pair. Specifically, a binary NIfTI mask was created to identify and mark regions of overlap, delineating the areas in the MRI images that aligned spatially with their corresponding CT images. This procedure was essential because segmentations generated from high-quality CT data were transferred onto MRI scans. Subsequently, this binary intersection mask was applied to trim the AI-predicted segmentations on both MRI and CT images within the test dataset, as well as the ground truth derived from manual skull segmentations performed on the CT scans. These intersected volumes served as the basis for evaluating and calculating segmentation accuracy.

### 2.6. Statistical Analysis

Segmentation performance was evaluated by comparing the manual CT segmentation with the AI segmentation for CT images and the AI segmentation for MRI images. Several metrics were employed that assess both volume and surface accuracy, including the Dice Similarity Coefficient (DSC), Mean Surface Distance (MSD), and Hausdorff Distance (HD), with all results reported as mean ± standard deviation. Table 1 provides detailed descriptions and mathematical formulations for each of the metrics utilized in this analysis. Statistical analyses were performed using Python 3.9.0.

### 2.7. Institutional Review Board Statement

The study was conducted following ethical guidelines and was approved by the Ethics Committee of Northwestern and Central Switzerland (EKNZ BASEC 2023–00446). Under the project titled “CT and MRI Segmentation for Detection and Classification of Diseases or Healthy Aging in Radiology” the approval was granted within the framework of using health-related personal data for research purposes, as outlined in Article 34 of the Swiss Human Research Act (HFG). All data were anonymized to ensure adherence to data protection regulations.

## 3. Results

When evaluating MRI sequences separately, the AI model trained and tested on individual sequence types (T1, T2, or FLAIR) yielded segmentation accuracy values that were very similar to those of the unified model trained on the combined dataset. In some cases, performance of the sequence specific models was slightly lower than that of the unified model. This finding suggests that the use of a single model capable of handling multiple MRI sequence types does not compromise accuracy and allows the inclusion of a greater volume of training data.

The accuracy of manual skull segmentation on CT images was compared to artificial intelligence-based segmentation results for both CT and MRI modalities. As shown in Table 2, AI segmentation performance on CT images significantly surpassed that on MRI across all evaluated metrics. Specifically, CT-based segmentation demonstrated higher Dice Similarity Coefficient (DSC) values (0.981 ± 0.004) compared to MRI (0.864 ± 0.035), indicating superior volumetric overlap. Surface-based metrics also favored CT, exhibiting substantially lower Mean Surface Distance (MSD: 0.078 ± 0.036 mm vs. 0.802 ± 0.301 mm), and Hausdorff Distance (HD: 6.785 ± 5.651 mm vs. 18.901 ± 13.479 mm).

**Figure 3:**
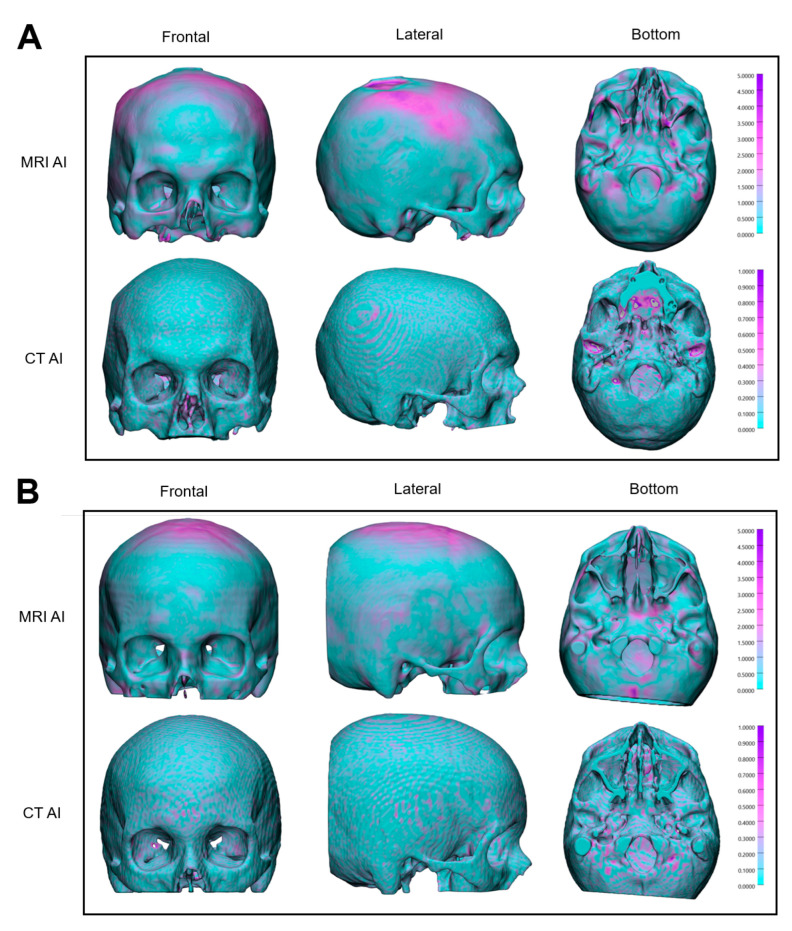
Surface deviation maps of AI-based skull segmentations from MRI (**top**) and CT (**bottom**) for two representative cases (**A**,**B**). Frontal, lateral, and bottom views show distance to the ground truth in mm, with color gradients indicating segmentation accuracy.

For better visualization of the deviations and understanding of the results, comparative distance maps between the AI-generated segmentations for MRI and CT images and the ground truth were produced (Figure 3). Color gradients represent surface deviations, ranging from −1.0 mm to +1.0 mm for CT AI segmentations and from −5.0 mm to +5.0 mm for MRI AI segmentations.

## 4. Discussion

The application of MRI in virtual surgical planning (VSP) for cranio-maxillofacial procedures is an appealing alternative to computed tomography (CT), particularly in pediatric and radiation-sensitive patients. However, MRI’s lower contrast resolution for bone structures poses a significant limitation. Unlike CT, which provides well-defined Hounsfield Units for bone, MRI lacks a standardized intensity scale, leading to difficulties in distinguishing bone from adjacent soft tissue. Although specialized sequences like Black Bone MRI have been developed to improve bone-to-soft-tissue contrast, their implementation remains technically demanding and not widely available. The results of this study suggest that, with the use of AI and CT-based mapping, these specialized sequences may no longer be necessary.

In our approach, high-resolution skull segmentations were initially derived from CT data and subsequently transferred onto the corresponding MRI scans through accurate multimodal registration. This overlap technique enabled the enrichment of the MRI dataset with anatomical detail that would otherwise not be discernible in MRI alone. Since CT provides superior bone contrast and spatial resolution, the CT-based segmentation introduced highly precise bone contours into the MRI domain. This not only increased the anatomical accuracy of the training data for the AI model but also led to significantly more detailed segmentations on MRI than would have been possible through direct manual annotation on MRI images alone. Essentially, information that is native to CT but absent in MRI was made accessible within the MRI space, enhancing segmentation detail without additional radiation exposure.

Despite these advantages, segmentation accuracy remains lower on MRI than on CT across all evaluated metrics. The mean Dice Similarity Coefficient (DSC) for AI-based MRI segmentation was 0.864 ± 0.035, compared to 0.981 ± 0.004 for CT. Surface-based accuracy measures such as Mean Surface Distance (MSD), and Hausdorff Distance (HD) also demonstrated the superiority of CT-based segmentation. Notably, the highest segmentation errors occurred in the maxillary region, where anatomical completeness in MRI was frequently limited. Standard MRI protocols often fail to cover both the skull and mandible in a single scan, leading to a lack of image data in the midface. This incomplete coverage directly impacts segmentation quality, particularly in complex anatomical areas such as the maxilla.

Compared to Zimmerman et al. (2021) [5], who segmented the cranial vault using a multi-atlas segmentation pipeline on bone-selective MRI (dual-echo UTE sequences), our study reported slightly lower segmentation accuracy for the MRI AI model (mean DSC 0.864 ± 0.035 vs. 0.91). Both studies used CT-based segmentations as the reference standard, registered to the corresponding MRI volumes to enable multimodal training and evaluation. The slightly lower Dice scores in our study are likely explained by the use of standard clinical MRI sequences (T1, T2, FLAIR), which offer reduced bone contrast and increased anatomical variability compared to bone-selective UTE imaging. However, to our knowledge, no previous study has developed or validated an AI-based method for cranial bone segmentation using conventional MRI. The only related work is the pilot study by Flügge et al. [25], which investigated automatic segmentation of teeth from T1-weighted MRI scans using a 3D nnU-Net model. Their model achieved a mean Dice score of 0.895, although segmentation accuracy was reduced in the presence of metal artifacts or anatomical abnormalities. In contrast, the present study proposes a novel methodology that leverages precise CT-to-MRI registration to generate anatomically accurate ground truth segmentations for training. This enables robust segmentation of osseous cranial structures directly from routine MRI, offering a viable alternative in clinical scenarios where CT acquisition is limited or contraindicated. In contrast, the present study trains and evaluates an AI model for skull segmentation on standard MRI using CT-derived labels obtained through accurate multimodal registration, enabling validation against high-quality ground truth rather than imprecise manual MRI segmentations. Conversely, the most accurate segmentation results were achieved in the frontal bone and orbital regions. These areas are typically well represented in standard MRI sequences and exhibit distinct structural boundaries that facilitate segmentation. This makes them particularly suitable for clinical applications such as orbital mirroring, patient-specific implant (PSI) design, or pre-bending of reconstruction plates and meshes, tasks that could potentially be completed without a CT scan. The segmentation accuracy observed in these structurally well-defined regions aligns with the tolerances typically required for virtual surgical planning. In clinical practice, deviations around 0.8 mm, such as the mean surface distance reported in this study, are considered acceptable for procedures including patient-specific implant design and orbital reconstruction. Although the overall Hausdorff Distance was influenced by outliers, these deviations were mainly found in areas with limited MRI coverage, such as the maxilla, and did not involve regions relevant to surgical planning. Therefore, the achieved accuracy in key anatomical areas supports the method’s applicability for selected virtual surgical planning scenarios. In clinical practice, MRI-based segmentation may be sufficiently accurate for tasks such as designing cutting guides or reconstructing the frontal bone and orbit, where our model performed best. This is particularly relevant in pediatric patients, where avoiding radiation is a priority. However, for high-precision applications like patient-specific implant design in complex regions, CT remains essential due to its superior spatial resolution and the high cost and accuracy demands of implant fabrication.

While this study demonstrates the feasibility of AI-driven skull segmentation from standard MRI, several limitations must be considered. First, small inaccuracies in the CT to MRI registration may occur, particularly in cases with low-resolution or poor-quality MRI scans, potentially affecting the transferred ground truth. To minimize this risk, all registrations were visually inspected by an experienced clinician in Materialise Mimics(version 25.0) software, where the anatomical overlap between CT and MRI could be assessed in multiplanar and three-dimensional views. Only cases with perfect overlap were included in the study. A considerable number of cases were therefore excluded, which reduced the final dataset size but ensured that registration errors did not propagate into the segmentation evaluation. Second, the dataset size was limited because it is inherently difficult to acquire paired CT and MRI scans from the same patient with sufficient anatomical overlap for accurate registration. Nevertheless, the present dataset of 200 paired scans is comparatively large for a proof of concept in this domain and provides a solid foundation for initial model development and validation. Efforts are currently underway to expand this work through multicenter studies that will include a larger and more heterogeneous dataset, thereby enabling broader validation. Furthermore, the MRI cohort contained heterogeneous sequences such as T1, T2, and FLAIR, which may introduce variability but also enhance the algorithm’s adaptability to real world clinical data.

An additional analysis was performed in which segmentation performance was evaluated separately for each MRI sequence type. Models trained and tested exclusively on T1, T2, or FLAIR sequences achieved segmentation accuracy values that were very similar to those of the unified model trained on all sequence types combined. In some cases, the sequence specific models produced slightly lower performance. For practical purposes, using a single model capable of processing multiple sequence types allows for a larger and more diverse training dataset, improves model robustness, and facilitates integration into clinical workflows where a variety of MRI sequences may be encountered.

Despite these strengths, segmentation performance remained lower on MRI than on CT (mean DSC 0.864 vs. 0.981), with larger surface deviations in complex regions, partly due to incomplete anatomical coverage, especially in the maxillary region.

Future developments should aim to train AI models on fully MRI-native datasets, standardize imaging protocols to improve consistency, and expand datasets across different scanners and field strengths. Moreover, refinement of surface accuracy and prospective clinical validation will be essential to enable reliable, radiation-free surgical planning in cranio-maxillofacial surgery. In specific trauma cases, pre-injury MRIs, if already available, could serve as a valuable basis for restoring previous anatomical configurations, supporting reconstructive planning without the need for additional imaging. Similarly, in oncologic cases, where MRI is already the preferred modality for evaluating soft tissue tumors, the ability to accurately segment bone structures could eliminate the need for supplementary CT scans. This dual-purpose use of MRI for both tumor and bone visualization significantly reduces radiation burden and simplifies diagnostic workflows.

## 5. Conclusions

This study presents a robust AI-based approach for skull segmentation on standard MRI, leveraging accurate CT-to-MRI registration to transfer high-resolution anatomical detail. While AI segmentation on CT remains more accurate, the MRI-based model substantially enhances segmentation quality without requiring ionizing radiation or specialized sequences such as Black Bone. The method demonstrates particular reliability in structurally well-defined regions, such as the frontal bone and orbit, supporting its use in trauma reconstruction, oncologic planning, and patient-specific implant design. By reducing manual workload and radiation exposure, this approach positions MRI as a viable alternative in selected cranio-maxillofacial applications. Importantly, the resulting segmentation quality is sufficient to serve as a foundation for virtual surgical planning, including the design of cutting guides and patient-specific implants, enabling a radiation-free and high-precision workflow that may redefine standards in cranio-maxillofacial surgery.

## Figures and Tables

**Table 1:** Metrics used in the study, with their formula and description.

Metric	Formula	Description
Dice similarity coefficient (DSC)	$DSC=\;\frac{2\left|A\cap B\right|}{\left|A\right|+\left|B\right|}=\frac{2\;TP}{2\;TP+FP+FN}$	The **Dice Similarity Coefficient (DSC)** evaluates segmentation quality by measuring the degree of overlap between two volumes, indicating their similarity.
Mean surface distance (MSD)	$MSD=\frac{1}{n_{A}+n_{B}}\left(\sum_{i=1}^{n_{A}}\underset{b\in B}{\min}{\left\|a_{i}-b\right\|}_{2}+\sum_{j=1}^{n_{B}}\underset{a\in A}{\min}{\left\|b_{j}-a\right\|}_{2}\right)$	The **Mean Surface Distance (MSD)** measures segmentation accuracy by computing the average of distances between surface points of the segmented volume and corresponding points on the ground truth surface, reflecting general segmentation precision.
Hausdorff distance (HD)	$\mathrm{HD}=\max{\{\sup}_{x\in X}d\left(x,Y\right),\sup_{y\in Y}d\left(X,y\right)\}$	The **Hausdorff Distance (HD)** quantifies segmentation accuracy by measuring the maximum distance between points on the segmented surface and their nearest counterparts on the ground truth, capturing the largest segmentation error.

**Table 2:** Comparison of AI segmentation results between MRI and CT imaging modalities. Data presented as mean ± standard deviation (SD) across metrics: Dice Similarity Coefficient (DSC), Mean Surface Distance (MSD), and Hausdorff Distance (HD). The arrow up means higher values are better, the arrow down that lower values are better.

	DSC ↑	MSD ↓	HD ↓
MRI	0.864 ± 0.035	0.802 ± 0.301	18.901 ± 13.479
CT	0.981 ± 0.004	0.078 ± 0.036	6.785 ± 5.651

## Data Availability

The original contributions presented in this study are included in the article. Further inquiries can be directed to the corresponding author.

## Abbreviations

The following abbreviations are used in this manuscript:

3D	Three-Dimensional
AI	Artificial Intelligence
CT	Computed Tomography
CMF	Cranio-Maxillofacial
DICOM	Digital Imaging and Communications in Medicine
DSC	Dice Similarity Coefficient
HD	Hausdorff Distance
HU	Hounsfield Unit
MRI	Magnetic Resonance Imaging
MSD	Mean Surface Distance
SD	Standard Deviation
STL	Standard Tessellation Language
VSP	Virtual Surgical Planning

## References

[1] Vyas K.S., Suchyta M.A., Hunt C.H. (2022). Black Bone MRI for Virtual Surgical Planning in Craniomaxillofacial Surgery. Semin. Plast. Surg..

[2] Eley K.A., Watt-Smith S.R., Golding S.J. (2017). “Black Bone” MRI: A novel imaging technique for 3D printing. Dentomaxillofacial Radiol..

[3] Chen H., Sprengers A.M.J., Kang Y. (2019). Automated segmentation of trabecular and cortical bone from proton density weighted MRI of the knee. Med. Biol. Eng. Comput..

[4] Schöckel L., Jost G., Seidensticker P. (2020). Developments in X-Ray Contrast Media and the Potential Impact on Computed Tomography. Investig. Radiol..

[5] Zimmerman C.E., Khandelwal P., Xie L., Lee H., Song H.K., Yushkevich P.A., Vossough A., Bartlett S.P., Wehrli F.W. (2022). Automatic Segmentation of Bone Selective MR Images for Visualization and Craniometry of the Cranial Vault. Acad. Radiol..

[6] Pearce M.S., Salotti J.A., Little M.P., McHugh K., Lee C., Kim K.P., Howe N.L., Ronckers C.M., Rajaraman P., Craft A.W. (2012). Radiation exposure from CT scans in childhood and subsequent risk of leukaemia and brain tumours: A retrospective cohort study. Lancet.

[7] Shah N., Bansal N., Logani A. (2014). Recent advances in imaging technologies in dentistry. World J. Radiol..

[8] Bornstein M.M., Horner K., Jacobs R. (2017). Use of cone beam computed tomography in implant dentistry: Current concepts, indications and limitations for clinical practice and research. Periodontology 2000.

[9] Whaites E., Drage N. (2020). Essentials of Dental Radiography and Radiology.

[10] Ludwig U., Eisenbeiss A.-K., Scheifele C., Nelson K., Bock M., Hennig J., Von Elverfeldt D., Herdt O., Flügge T., Hövener J.-B. (2016). Dental MRI using wireless intraoral coils. Sci. Rep..

[11] Flügge T., Hövener J.-B., Ludwig U., Eisenbeiss A.-K., Spittau B., Hennig J., Schmelzeisen R., Nelson K. (2016). Magnetic resonance imaging of intraoral hard and soft tissues using an intraoral coil and FLASH sequences. Eur. Radiol..

[12] Burian E., Probst F.A., Weidlich D., Cornelius C.-P., Maier L., Robl T., Zimmer C., Karampinos D.C., Ritschl L.M., Probst M. (2020). MRI of the inferior alveolar nerve and lingual nerve—Anatomical variation and morphometric benchmark values of nerve diameters in healthy subjects. Clin. Oral Investig..

[13] Cankar K., Vidmar J., Nemeth L., Sersa I. (2020). T2 mapping as a tool for assessment of dental pulp response to caries progression: An in vivo MRI study. Caries Res..

[14] Juerchott A., Pfefferle T., Flechtenmacher C., Mente J., Bendszus M., Heiland S., Hilgenfeld T. (2018). Differentiation of periapical granulomas and cysts by using dental MRI: A pilot study. Int. J. Oral Sci..

[15] Algarín J.M., Díaz-Caballero E., Borreguero J., Galve F., Grau-Ruiz D., Rigla J.P., Bosch R., González J.M., Pallás E., Corberán M. (2020). Simultaneous imaging of hard and soft biological tissues in a low-field dental MRI scanner. Sci. Rep..

[16] Bracher A.-K., Hofmann C., Bornstedt A., Hell E., Janke F., Ulrici J., Haller B., Geibel M.-A., Rasche V. (2013). Ultrashort echo time (UTE) MRI for the assessment of caries lesions. Dentomaxillofacial Radiol..

[17] Stumpf K., Kaye E., Paul J., Wundrak S., Pauly J.M., Rasche V. (2020). Two-dimensional UTE overview imaging for dental application. Magn. Reson. Med..

[18] Weiger M., Pruessmann K.P., Bracher A.-K., Köhler S., Lehmann V., Wolfram U., Hennel F., Rasche V. (2012). High-resolution ZTE imaging of human teeth. NMR Biomed..

[19] Idiyatullin D., Corum C., Moeller S., Prasad H.S., Garwood M., Nixdorf D.R. (2011). Dental magnetic resonance imaging: Making the invisible visible. J. Endod..

[20] Hoff M., Andre J., Stewart B., Saba L. (2016). Artifacts in magnetic resonance imaging. Image Principles, Neck, and the Brain.

[21] Wang S., Pang X., de Keyzer F., Feng Y., Swinnen J.V., Yu J., Ni Y. (2023). AI-based MRI auto-segmentation of brain tumor in rodents, a multicenter study. Acta Neuropathol. Commun..

[22] Nielsen J.D., Madsen K.H., Puonti O., Siebner H.R., Bauer C., Madsen C.G., Saturnino G.B., Thielscher A. (2018). Automatic skull segmentation from MR images for realistic volume conductor models of the head: Assessment of the state-of-the-art. Neuroimage.

[23] Vinayahalingam S., Berends B., Baan F., Moin D.A., van Luijn R., Bergé S., Xi T. (2023). Deep learning for automated segmentation of the temporomandibular joint. J. Dent..

[24] Isensee F., Jaeger P.F., Kohl S.A.A. (2021). nnU-Net: A self-configuring method for deep learning-based biomedical image segmentation. Nat. Methods.

[25] Flügge T., Vinayahalingam S., van Nistelrooij N., Kellner S., Xi T., van Ginneken B., Bergé S., Heiland M., Kernen F., Ludwig U. (2025). Automated tooth segmentation in magnetic resonance scans using deep learning—A pilot study. Dentomaxillofac. Radiol..

